# Computed Tomographic Sarcopenia in Pancreatic Cancer: Further Utilization to Plan Patient Management

**DOI:** 10.1007/s12029-021-00672-4

**Published:** 2021-07-22

**Authors:** Mustafa Jalal, Jennifer A. Campbell, Jonathan Wadsley, Andrew D. Hopper

**Affiliations:** 1 Academic Department of Gastroenterology, Royal Hallamshire Hospital, Glossop Road, Sheffield, S10 2JF UK; 2 Department of Oncology, Weston Park Hospital, Whitham Road, Sheffield, S10 2SJ UK

**Keywords:** Pancreatic cancer, Skeletal muscle, Sarcopenia, Palliative chemotherapy, Best supportive care

## Abstract

**Purpose:**

The presence of a sarcopenia adversely affects the prognosis of patients with pancreatic cancer. There is an emerging role for using computed tomography (CT) to calculate skeletal muscle index (SMI) and the presence of sarcopenia. The aim of this study was to assess if detecting ‘computed tomographic sarcopenia’ is feasible and can contribute to the management of patients with locally advanced pancreatic cancer (LAPC).

**Methods:**

Patients diagnosed with LAPC referred for endoscopic ultrasound-guided biopsy (EUS-B) by our regional cancer network were identified. Age, body mass index (BMI), and Eastern Cooperative Oncology Group performance status (ECOG-PS) were noted. CT images were analysed for SMI and the presence of sarcopenia. Decision outcomes on receiving chemotherapy or not were collected from the regional oncology database.

**Results:**

In total, 51/204 (25%) patients with LAPC who underwent EUS-B were not given chemotherapy and received best supportive care (BSC) only. The prevalence of sarcopenia (*p* = 0.0003), age ≥ 75 years old (*p* = 0.03), and ECOG-PS 2–3 (*p* = 0.01) were significantly higher in the patients receiving BSC only. Logistic regression analysis demonstrated that SMI was the only independent associated factor identifying patients with LAPC who were treated with BSC only and not chemotherapy after adjusting for age and ECOG-PS.

**Conclusion:**

Our study has shown that computed tomographic skeletal muscle analysis at the time of a diagnostic CT for patients with pancreatic cancer is feasible and can detect sarcopenia and malnourished patients who are much less likely to take up chemotherapy. These patients could be triaged to oncology assessment prior to EUS-B to avoid unnecessary investigations.

## Introduction

Pancreatic adenocarcinoma (PDAC) is the 11th most common cancer in the UK, and it accounts for 3% of cancer cases per year [1]. PDAC has a poor prognosis with 80% of patients presenting with local or advanced disease at diagnosis [2]. Recent reports have demonstrated that the loss of skeletal muscle mass during neo-adjuvant chemotherapy for borderline resectable pancreatic cancer was associated with poor survival and lower resection rates [3, 4]. A low skeletal muscle mass and strength are defined as sarcopenia [5]. Sarcopenia has a reported prevalence of 25–63% in PDAC, and it has been shown to impact adversely on the prognosis of patients undergoing surgical resection or palliative therapy [6–11]. Sarcopenia can still occur in patient with a raised body mass index (BMI), and the combination of both is given the term ‘sarcopenic obesity’ which was shown to adversely impact on survival in PDAC [6].

Given its aggressive course, pancreatic cancer has been described as a ‘medical emergency’, and patients undergo a rapid sequence of tests to stage and confirm the diagnosis once made [12]. Unfortunately, only 8% of patients present with resectable pancreatic cancer; therefore, the remaining are considered for palliative chemotherapy only [1, 13]. For metastatic or locally advanced pancreatic cancer (LAPC) disease, endoscopic ultrasound-guided biopsy (EUS-B) is recommended prior to palliative chemotherapy [14–16]. However, the availability of EUS-B services is often limited to tertiary centres and university hospitals requiring some patients to travel significant distances for an invasive procedure that requires significant sedation [17]. It has been recorded by cancer support charities that, in all patients with local and metastatic advanced cancer who undergo a biopsy from the primary or a metastatic site, only 28% of patients go on to receive chemotherapy [18]. Therefore, an EUS-B can be an unnecessary and onerous procedure for many patients with PDAC and delay them receiving supportive and beneficial treatments that focus on nutrition, pain, and psychological support termed best supportive care (BSC) [13, 19, 20].

There is an emerging role for combining computed tomography (CT) scan with image analysis software to measure body composition and assess presence of sarcopenia [21]. Patients undergo CT scanning as part of the standard care for suspected PDAC [22] and, therefore, presents the opportunity to gain prognostic information about a patient. The aim of this study was to assess if presence of sarcopenia on the diagnostic CT scan could predict patients who do not go on to receive palliative chemotherapy in LAPC after a EUS-B.

## Methods

Prospective identification of patients with diagnosis of LAPC referred for EUS-B by the regional cancer network between Jan 2016 and Dec 2018. Patient information collected included the following: sex, age, BMI, and Eastern Cooperative Oncology Group performance status (ECOG-PS). Decision outcomes on receiving chemotherapy or not were collected from the regional oncology database.

### Computerised Tomography Skeletal Muscle Mass Measurements

Diagnostic CT images (Canon Aquilion One Scanners, 120 kV, slice thickness 1 mm) using intravenous contrast and examined in the venous phase were analysed for sarcopenia. Skeletal muscle was analysed from a single axial CT image at the level of the third lumbar vertebra using a commercially available software (sliceOmatic V5, Tomovision, Quebec, Canada). Images were anonymised, given a study number, and downloaded in Digital Imaging and Communications in Medicine (DICOM) format.

**Fig. 1:**
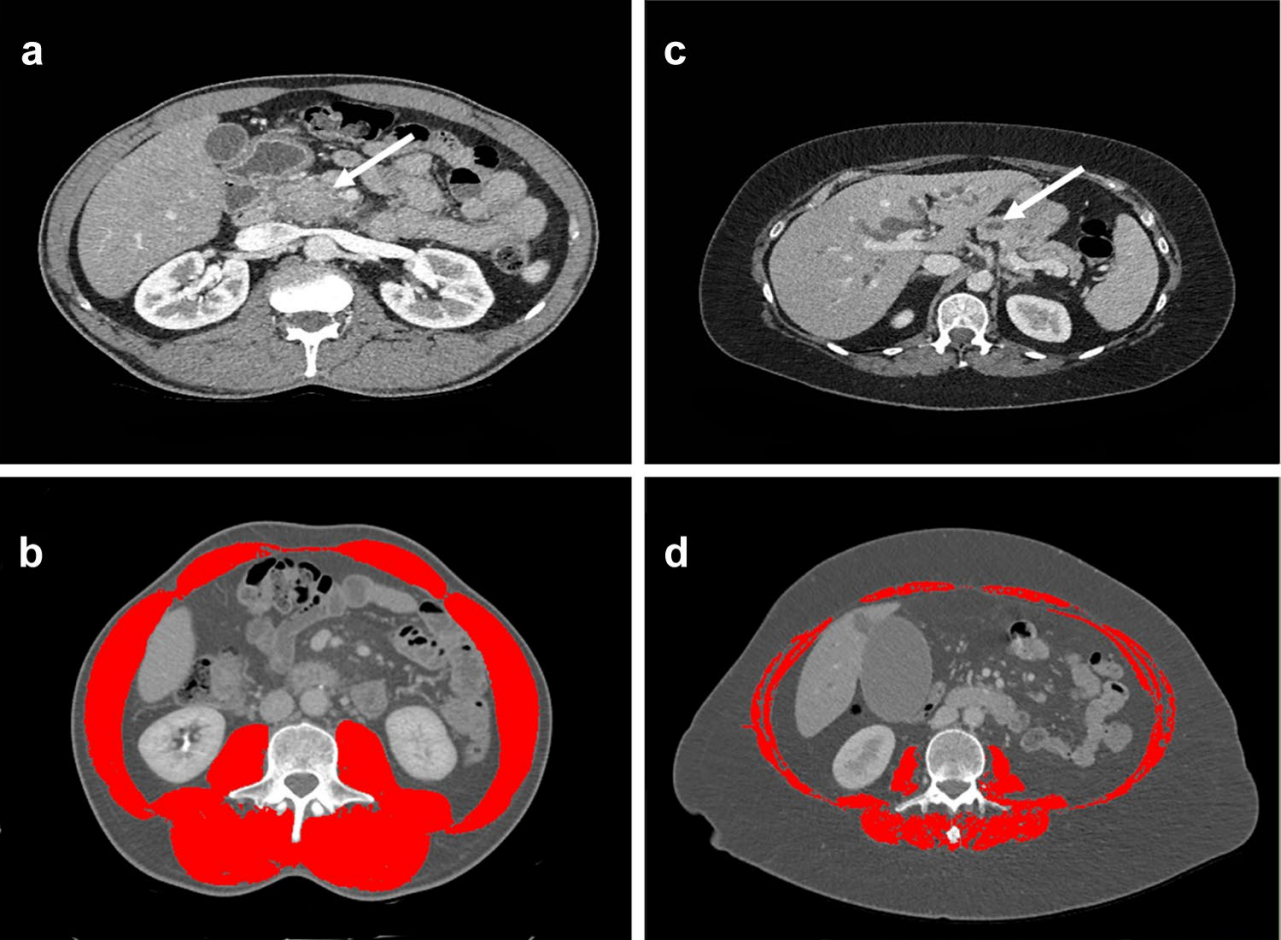
Computed tomographic skeletal muscle analysis in patients with locally advanced pancreatic cancer. Patient 1: **a** locally advanced tumour visible in the uncinate process (white arrow), despite patient's relatively low BMI (21.6 kg/m^2^) computed tomographic muscle mass analysis at the L3 level, (**b**) showed a normal ratio of skeletal muscle (highlighted red) SMI 50.1 cm^2^/m^2^ with the patient receiving chemotherapy. Patient 2: **c** with a locally advanced pancreatic cancer in the head and a dilated main pancreatic duct (white arrow), despite the high BMI 32.1 kg/m^2^ computed tomographic muscle mass analysis demonstrated a low SMI 31.3 cm^2^/m^2^ consistent with sarcopenia, the patient did not go on to receive chemotherapy. The presence of a high BMI > 25 kg/m^2^ and sarcopenia is termed ‘sarcopenic obesity’ [24]

Using sliceOmatic, skeletal muscle area was identified using Hounsfield unit (HU) thresholds (–29 to + 150) [8]. Cross-sectional area (cm^2^) of skeletal muscle (psoas, erector spinae, quadratus lumborum, transversus abdominis, external and internal oblique abdominis, and rectus abdominis muscles) was measured and identified as shown in Fig. 1. The surface area of skeletal muscle was normalised using the square of patient’s height to calculate skeletal muscle index (SMI) expressed as (cm^2^/m^2^). Sarcopenia was defined as a SMI < 41 cm^2^/m^2^ for women, and SMI < 43 cm^2^/m^2^ if BMI < 25 kg/m^2^ or < 53 cm^2^/m^2^ if BMI ≥ 25 kg/m^2^ for men [23].

Using previously accepted definitions, sarcopenic obesity in this study was referred to the presence of both sarcopenia and a BMI ≥ 25 kg/m^2^ [24].

### Statistical Analysis

Fischer’s exact test and Mann Whitney *U* test were used to compare categorical and continuous variables. Binary logistic regression was used to assess association of variables with non-uptake of chemotherapy. A *p* value < 0.05 was considered statistically significant. Statistical analyses were performed using SPSS (IBM SPSS statistics version 25, Chicago, Illinois, USA). The study was approved by local research ethics committee (IRAS 301,193; STH 21,885).

## Results

A total of 204 patients with LAPC referred for EUS-B were included, and 114 males with median age 69 (42–84) years. The site of the cancers identified was recorded in the head 66.2% (135), body 30.4% (62), and tail 3.4% (7) of the pancreas.

Sarcopenia was present in 54.4% (111) patients and was significantly higher in female 63.3% compared to male patients 47.4%, *p* = 0.03. Sarcopenic obesity was present in 27% (55). The majority of patients had PS ≤ 1 85.8% (175) compared to PS ≥ 2 14.2% (29).

Of the 204 patients recruited, 75% (153) received at least one dose of a chemotherapy agent compared to 25% (51) who had BSC (Table 1). SMI was significantly lower in the BSC group compared to chemotherapy group. The presence of sarcopenia, age ≥ 75 years old, and ECOG-PS 2–3 were more common in BSC. There were no significant differences in comparing the site of cancer, BMI, BMI < 20 kg/m^2^, or sarcopenic obesity between the BSC and chemotherapy groups.

**Table 1:** Comparison of the demographics and anthropometric assessments for patients with locally advanced pancreatic cancer receiving best supportive care only or chemotherapy

Parameter	Best supportive care% (*n*) or median (range)	Chemotherapy% (*n*) or median (range)	*P* Value
Number of patients	25% (51)	75% (153)	
Age, years	71 (49–84)	68 (42–83)	***0.02***
Age ≥ 75 years	37.3% (18)	19.6% (30)	***0.03***
Male	45.1% (23)	59.5% (91)	NS
Female	54.9% (28)	40.5% (62)	
ECOG-PS 0–1	74.5% (38)	89.5% (137)	***0.01***
ECOG-PS 2–3	25.5% (13)	10.5% (16)	
BMI kg/m^2^	23.6 (17.7–42.2)	25 (15.4–50.8)	NS
BMI < 20 kg/m^2^	15.7% (8)	8.5% (13)	NS
Sarcopenia	76.5% (38)	47.8% (73)	***0.0003***
SMI	40.5 (26.3–60)	44.2 (26.5–80.9)	***0.02***
Sarcopenic obesity	25.5% (13)	27.5% (42)	NS

*NS* not significant, *ECOG-PS* Eastern Cooperative Oncology Group performance status, *BMI* body mass index, *SMI* skeletal muscle index

Performing logistic regression analysis demonstrated that SMI was the only associated factor identifying patients to choose BSC (*p* = 0.04) and not uptake chemotherapy after adjusting for age, and ECOG-PS. The estimated odds ratio favoured a decrease of nearly 4.5% for opting into BSC for every 1-unit increase of SMI.

## Discussion

We have shown that SMI measurement at the time of a diagnostic CT for pancreatic cancer is feasible and can identify sarcopenia. We have also shown that the presence of ‘computed tomographic sarcopenia’ objectively identifies malnourished patients who are much less likely to take up chemotherapy. To our knowledge, the association between sarcopenia and low uptake of palliative chemotherapy in patients with LAPC has not been described before and could facilitate a patient’s care pathway. The average time for SMI measurement for a single patient was 2.5 min so could make up part of an initial clinical or multidisciplinary team assessment.

A notable benefit of SMI assessment for patients with PDAC from the diagnostic CT is that it performs an early, objective nutritional assessment tool. Patients presenting with suspected malnutrition can be assessed with alternative methods such as percentage weight loss [25], but a single BMI measurement is of limited diagnostic use on its own [26]. Malnutrition screening tools are available which do have a high inter-rater reliability (*κ* = 0.67–1.00) [27–29], and recording specific anthropometric measurements is possible with serial readings [30, 31]. However, alternative nutritional assessment methods should also be interpreted with caution in an obese population [31, 32]: our study showed that nearly half of our patients were overweight, and approximately 1 in 4 patients had sarcopenic obesity. The average weight-losing patient in PDAC is overweight which highlights the reported link with obesity [6, 33]. A further advantage of sarcopenia assessment is that patients with sarcopenic obesity may be at higher risk of chemotherapy toxicity due to dosing currently being based on body surface area which does not take into account body composition [22]. The benefits of nutritional assessment and subsequent intervention have been demonstrated in one study showing an independent association with survival among patients with unresectable pancreatic cancer (hazard ratio 2.12) regardless of chemotherapy treatment [19].

Despite tissue diagnosis, 51 (25%) of our patients with LAPC did not receive chemotherapy despite undergoing a procedure to obtain a biopsy specifically to be considered for this purpose. Although still high, this proportion is much lower than other reported data would predict [1], and lower than our initial data collection published in abstract form [34]. Our findings would be supported by our exclusion of patients with metastatic disease (who would undergo liver biopsy locally) and our cancer network awareness of the initial abstract findings prompting attempts to improve patient assessment prior to EUS-B referral.

The subjective assessment of patients ECOG-PS is challenging and can alter quickly; in this study, it proved to be a limitation, as we did not eliminate the inter-observer variability of ECOG-PS; and this is in contrast to computed tomographic SMI assessment which has been shown to have a good intra-observer agreement [21, 35, 36]. ECOG-PS is an important functional assessment for sarcopenia and can be combined with computed tomographic skeletal muscle analysis if required. Another limitation is the association between sarcopenia and increasing age [37]; however, the regression analysis identified SMI only to be associated with non-uptake of chemotherapy.

Our study has shown that performing SMI measurement at the time of a diagnostic CT for pancreatic cancer is feasible and can identify sarcopenia and malnourished patients who are much less likely to take up chemotherapy. This information along with ECOG-PS and age could help predict patients that are unlikely to take up palliative chemotherapy in LAPC. These patients could be triaged to initial oncology assessment for nutritional assessment prior to EUS-B referral to gain nutritional support and best supportive care and avoid unnecessary investigations.

## Data Availability

The datasets used during the present study are available from the corresponding author upon reasonable request.
